# Suppression of Bedbug’s Reproduction by RNA Interference of Vitellogenin

**DOI:** 10.1371/journal.pone.0153984

**Published:** 2016-04-20

**Authors:** Minoru Moriyama, Takahiro Hosokawa, Masahiko Tanahashi, Naruo Nikoh, Takema Fukatsu

**Affiliations:** 1 Bioproduction Research Institute, National Institute of Advanced Industrial Science and Technology (AIST), Tsukuba, Japan; 2 Faculty of Science, Kyushu University, Fukuoka, Japan; 3 Department of Liberal Arts, The Open University of Japan, Chiba, Japan; 4 Department of Biological Sciences, Graduate School of Science, University of Tokyo, Tokyo, Japan; 5 Graduate School of Life and Environmental Sciences, University of Tsukuba, Tsukuba, Japan; New Mexico State University, UNITED STATES

## Abstract

Recent resurgence of the bedbug *Cimex lectularius* is a global problem on the public health. On account of the worldwide rise of insecticide-resistant bedbug populations, exploration of new approaches to the bedbug control and management is anticipated. In this context, gene silencing by RNA interference (RNAi) has been considered for its potential application to pest control and management, because RNAi enables specific suppression of target genes and thus flexible selection of target traits to be disrupted. In this study, in an attempt to develop a control strategy targeting reproduction of the bedbug, we investigated RNAi-mediated gene silencing of vitellogenin (Vg), a major yolk protein precursor essential for oogenesis. From the bedbug transcriptomes, we identified a typical *Vg* gene and a truncated *Vg* gene, which were designated as *ClVg* and *ClVg-like*, respectively. *ClVg* gene was highly expressed mainly in the fat body of adult females, which was more than 100 times higher than the expression level of *ClVg*-like gene, indicating that *ClVg* gene is the primary functional *Vg* gene in the bedbug. RNAi-mediated suppression of *ClVg* gene expression in adult females resulted in drastically reduced egg production, atrophied ovaries, and inflated abdomen due to hypertrophied fat bodies. These phenotypic consequences are expected not only to suppress the bedbug reproduction directly but also to deteriorate its feeding and survival indirectly via behavioral modifications. These results suggest the potential of *ClVg* gene as a promising target for RNAi-based population management of the bedbug.

## Introduction

An important factor that makes insects serious pests is their high reproductive ability. Hence, understanding how insects produce plentiful offspring and how the reproductive capability can be suppressed have been among major subjects in researches on pest control and management [1–3]. In most insects, as in oviparous animals in general, females deliver a considerable amount of nutritious resources to their eggs, which is mainly mediated by a major yolk precursor protein called vitellogenin (Vg) [4–7]. Vg protein, a member of the large lipid transfer protein superfamily [8], is mainly synthesized in the fat body and subjected to a variety of post-translational processing. Typically, Vg protein is proteolytically cleaved at particular sites, and the subunits are assembled together with lipids, carbohydrates and other nutrients, thereby forming a large oligomeric phosphoglycolipoprotein [6,7]. The complex is subsequently secreted into hemolymph and taken up by growing oocytes through endocytosis mediated by Vg receptors (VgR) [5].

The common bedbug *Cimex lectularius* (Hemiptera: Cimicidae) is a nuisance pest that feeds on blood of humans and other warm-blooded animals [9,10]. Although the bedbugs do not transmit fatal disease agents of humans, their bites annoy people by causing cutaneous manifestations, urticarial reactions and, occasionally, anaphylaxis [11]. The domestic infestation of this nuisance pest may result in adverse effects on mental health of residents, such as emotional distress, anxiety, insomnia and paranoia [12]. A single female bedbug can produce 200–500 eggs during its lifetime, which underlies its rapid population growth once infestation occurs [13]. The recent resurgence of the bedbug across the world, which might have been facilitated by increasing international travel and trade, is regarded as a global problem on public health [10,11]. Conventional eradication means are nowadays not effective, mainly because of the rise of insecticide-resistant populations of the bedbug [12]. Therefore, exploration of new approaches to the bedbug control and management is of urgent need.

In this context, gene silencing by RNA interference (RNAi) has recently attracted much attention not only in characterizing gene functions but also in controlling insect pests [14–17]. The high specificity of RNAi machinery against target nucleotide sequences conceptually enables eradication programs with minimal ecological side effects. It can also provide an alternative strategy for pest management without direct lethal actions, in which key genes responsible for pest status including fecundity, ability to utilize peculiar plant/animal hosts, pathogen transmitting capacity, resistance to chemical pesticides, etc., are targeted [14,18,19].

In diverse pest arthropods including cockroaches, fire ants and ticks, previous studies reported successful instances of RNAi targeting *Vg* gene [20,21] or *VgR* gene [22–24], which generally caused negative consequences in the ovarial development. Several studies reported that RNAi works in the bedbug, although those studies are not intended to population control of the nuisance pest [25,26]. In this study, in an attempt to develop a novel approach to reproduction control of the bedbug, we investigated *Vg* genes as a target of RNAi-mediated gene silencing.

## Materials and Methods

### Insect

A laboratory strain of the bedbug JESC, which had been maintained at the Japan Environmental Sanitation Center for decades [27], was used in this study. Adults and nymphs were kept in plastic Petri dishes (9 cm in diameter and 2 cm in depth) with several pieces of pleated filter paper (2 cm x 3 cm) at 25°C under constant darkness. The insects were fed once a week on commercially-purchased rabbit blood (Kohjin Bio, Japan) warmed at 35–36°C using a membrane feeding system as described previously [27].

### Identification of Bedbug *Vg* Genes

*Vg* genes of the bedbug were surveyed in the expression sequence tag (EST) database [28] and the RNA sequencing (RNAseq) database [29]. The short RNAseq reads (SRA accession number, SRP008480) were assembled using Trinity v2.0.2 [30]. Using BLASTX similarity searches against the Uniprot protein database (www.uniprot.org), we obtained partial fragments of the *Vg* genes. Then, full-length transcript sequences were obtained by PCR amplification and DNA sequencing using the primers listed in Table 1 and the bedbug cDNA libraries constructed previously [28]. The *Vg* gene sequences determined in this study were deposited in the DNA Data Bank of Japan with the accession numbers LC115022 and LC115023.

**Table 1:** Primers used in this study.

Name	Gene	Sequence (5’ to 3’)	Usage^a^
ClVg_235_F	*ClVg*	GGA AAA CTC ACC GTC CAA CC	cloning
ClVg_370_R	*ClVg*	TTG TTG GAA AAG GGG AGT TG	cloning
ClVg_1660_F	*ClVg*	CCA TAC TCC AAA GCA TCA CC	cloning
ClVg_1686_R	*ClVg*	CGT TCG GCA TCA GAA GTG	cloning
ClVg_3341_R	*ClVg*	TTC GAC ATC AAC ATC GAC AC	cloning
ClVg_4021_F	*ClVg*	CGA TTC CTC TTC TTC GTG AG	cloning
ClVg_4106_R	*ClVg*	AAG TGG GAC ATT GGG TGA AG	cloning
ClVg_4501_R	*ClVg*	TCA AAC CTG ACA ACG ACA TCT AC	cloning
ClVg_4634_F	*ClVg*	TCC ACC CAC ATT ACA ACA CC	cloning/dsRNA
ClVg_4942_R	*ClVg*	GCT GTC TGT CCG TTG ACC TT	cloning/dsRNA
ClVg_5342_F	*ClVg*	ACA GCC GCA AAT CAC AAC AC	qPCR
ClVg_5474_R	*ClVg*	GGC ACT CGG GCA TCT TTC	qPCR
ClVgL_13_F	*ClVg-like*	ATT TTT AAT CGT CCC GCC GC	cloning
ClVgL_83_R	*ClVg-like*	AGG GAC GAA TGA TCC ATC GC	cloning
ClVgL_1380_F	*ClVg-like*	ACA ACG AGA CAG TGT CAT CCC	cloning
ClVgL_1431_R	*ClVg-like*	TGG TTT TCA AGC CTC CCA TG	cloning
ClVgL_2691_F	*ClVg-like*	TGC ATA CTC AAG CAG TCC GG	cloning
ClVgL_2778_R	*ClVg-like*	GCA ACG AAA CCT GGA AAG GC	cloning
ClVgL_4033_F	*ClVg-like*	ACC GAC AAG ATG AGC AGA GC	cloning/pPCR
ClVgL_4119_R	*ClVg-like*	TGC AAG AGC GAG TTT GAT CG	cloning/pPCR
ClEF1alpha_F^b^	*Ef1α*	TGG TAT CGA CAA ACG TAC CAT C	qPCR
ClEF1alpha_R^b^	*Ef1α*	GCT CGG CCT TGA GCT TGT C	qPCR

^a^ Primers were designed for the following purposes: cloning, *Vg* gene sequence identification; dsRNA, gene fragment amplification for dsRNA synthesis; qPCR, gene expression quantification by RT-qPCR.

^b^ These primers were originated from Moriyama et al. [28].

### Molecular Phylogenetic and Evolutionary Analyses

Multiple alignment of Vg protein sequences was conducted using MUSCLE program [31]. Phylogenetic trees were constructed by maximum-likelihood and neighbor-joining methods using MEGA ver. 6.0 with 1,000 bootstrap replications [32]. Relative rate tests were performed using RRTree [33].

### Quantification of *Vg* Gene Expression

Reverse-transcription quantitative PCR (RT-qPCR) was conducted for evaluating expression levels of the *Vg* genes in different tissues of the bedbug essentially as described previously [28]. Adult females of 1–2 months after emergence were dissected in a phosphate buffered saline. Total RNA samples of the isolated tissues were purified and reverse-transcribed using RNAiso plus (TaKaRa), RNeasy columns (QIAGEN) and Improm II Reverse Transcription System (Promega), and subjected to RT-qPCR using the primers listed in Table 1. A standard curve was drawn using the PCR fragment cloned into the pT7 blue plasmid (Novagene). Expression levels of the *Vg* genes were normalized by quantifying expression levels of *elongation factor 1α* (*EF1α*) gene using the primers listed in Table 1. Each biologically independent sample was measured twice and averaged.

### RNAi Treatment and Phenotype Inspection

Double-stranded RNA (dsRNA) was synthesized from the PCR product of the *Vg* gene using MEGAscript RNAi kit (Ambion), in which fragment amplification was conducted using the gene specific primers (see Table 1) attached to T7 promoter sequence. For control experiments, dsRNA of *β-lactamase* gene fragment from pT7 blue plasmid was synthesized. Adult insects within two weeks after emergence were collected and assigned to each treatment group. Three females and three males were placed in each plastic petri dish (6 cm in diameter and 1.5 cm in depth) with two pieces of pleated filter paper, where they were allowed to mate freely. After two days from the first blood meal, the females were injected with 20 ng or 200 ng of dsRNA dissolved in 0.5 μl of distilled water from the basement membrane of a hind leg using a fine glass capillary needle. These bedbugs were fed once a week, when the number of eggs and hatched nymphs were recorded. Four weeks after the injection, these females were subjected to histological analysis or RNA extraction. The ovaries of some females were dissected out, photographed, and measured under a stereoscopic microscope and digital camera system (S8Apo and EC3, Leica Microsystems). Total RNA was extracted from the whole body, and expression levels of the *Vg* gene were quantified as described above.

### Statistics

Gene expression levels were compared by fitting to a generalized linear model (GLM) [34], where Gamma error structure with log link function was assumed. The following models were selected for each GLM analysis: binomial error structure with logit link function for survival and hatching rates; Gaussian error structure for oocyte size; and Poisson error structure with log link function for number of eggs. When sample overdispersion was observed, we adopted a generalized linear mixed model (GLMM) that considers individual variation as a random effect. If deviance reduction due to the treatment term was significant in chi-square test, we compared its effect between each treatment group by Tukey-type multiple comparisons. All statistical analyses were performed using R ver. 3.2 [35].

## Results

### Bedbug *Vg* and *Vg-like* Genes

**Fig 1:**
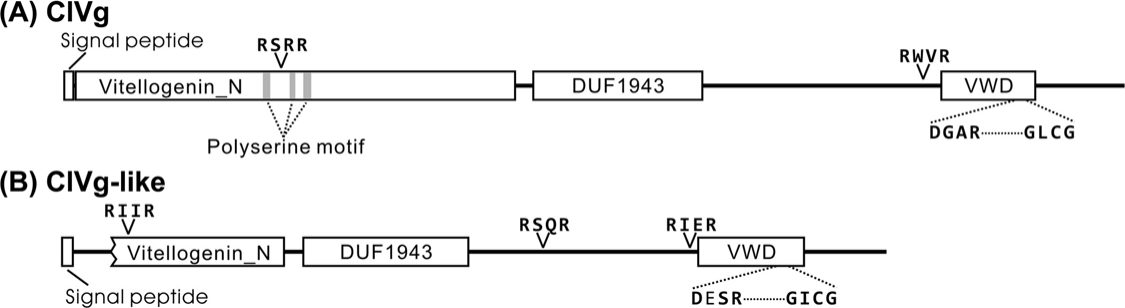
Schematic representation of protein structures encoded by vitellogenin genes, *ClVg* and *ClVg-like*, of the bedbug. (A) ClVg protein. (B) ClVg-like protein. The following domains typical of vitellogenin proteins are illustrated: Vitellogenin_N, a lipoprotein amino terminal region; DUF1943, a domain of unknown function consisting of several large open beta-sheets; VWD, a von Willebrand factor type D domain. The consensus RXXR cleavage sites and DGXR-GL/ICG motifs are also indicated.

From transcriptome databases of the bedbug [28,29], we obtained partial gene sequences exhibiting significant similarities to known *Vg* genes. By making use of the sequences, we cloned and identified full-length cDNA sequences of two putative *Vg* genes. Fig 1A shows the amino acid sequence (1,863 residues) deduced from the first *Vg* gene, which encoded all conserved structures typical of insect Vg proteins [6]. In addition to the well-conserved RXXR cleavage site flanked by polyserine motifs at the N-terminal region, this protein contained another RXXR cleavage site at the C-terminal region (Fig 1A), indicating possible multiple cleavages as known for Vg protein of the bean bug *Riptortus pedestris* [36]. The amino acid sequence inferred from the other *Vg* gene (1,449 residues) showed 36% sequence identity to the sequence of the former Vg protein. This sequence was somewhat diverged from those of conventional Vg proteins known from diverse insects, with a truncated lipid binding domain, so-called Vitellogenin_N, and without the polyserine motif (Fig 1B). In addition, there was an amino acid substitution in the well-conserved DGXR-GL/ICG motif at the C-terminal region [6]. Hereafter we refer to the former gene as *ClVg*, and the latter gene as *ClVg-like*.

**Fig 2:**
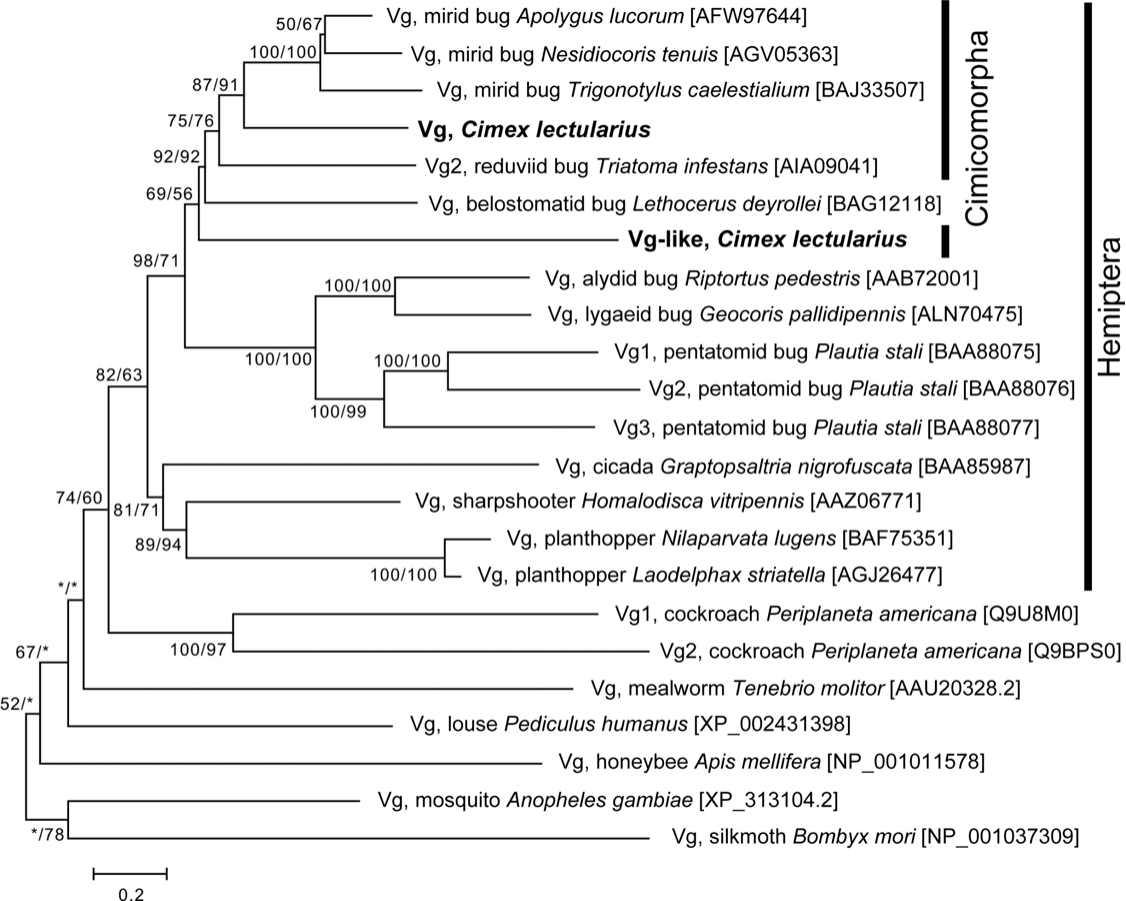
Phylogenetic placement of vitellogenin proteins, ClVg and ClVg-like, of the bedbug. The two Vg protein sequences of the bedbug (bold face) were analyzed with fourteen Vg protein sequences of hemipteran insects and seven Vg protein sequences of other insects, from which the N-terminal region lacking in ClVg-like was removed. Pairwise alignments of 1,387–1,562 amino acid sites were subjected to the analysis. A maximum-likelihood phylogeny is shown with bootstrap probabilities in the order of neighbor-joining/maximum likelihood. Asterisks indicate statistical values lower than 50%. The ranges of the infraorder Cimicomorpha and the order Hemiptera are shown on the right side.

In the NCBI non-redundant protein database, the ClVg protein was the most similar to Vg2 of *Triatoma infestans* (Hemiptera: Reduviidae) with 52% sequence identity, while the ClVg-like protein was the most similar to Vg of *Apolygus lucorum* (Hemiptera: Miridae) with 36% sequence identity. Molecular phylogenetic analyses were performed by including Vg protein sequences of hemipterans and other insects after removing the N-terminal region that was lacking in the ClVg-like protein (Fig 2). While both the ClVg protein and the ClVg-like protein were placed within the clade of the order Hemiptera, the ClVg-like protein was located outside the infraorder Cimicomorpha to which bedbugs, reduviid bugs, and mirid bugs belong. On the phylogeny, the ClVg-like branch was elongated (Fig 2), and relative rate tests showed accelerated molecular evolution in the lineage of *ClVg-like* gene (Table 2). Hence, although the ClVg-like protein was placed even outside of the belostomatid water bug *Lethocerus deyrollei* of the infraorder Nepomorpha, it seems doubtful that the *ClVg-like* gene diverged from the common ancestor of the Cimicomorpha and the Nepomorpha, considering the low resolution of the phylogeny and the accelerated molecular evolution in the lineage of the *ClVg-like* gene.

**Table 2:** Relative rate tests for comparing the molecular evolutionary rate of ClVg protein sequence of the bedbug with those of ClVg-like protein sequence and other Vg protein sequences of hemipteran insects based on 1,235 unambiguously aligned amino acid sites.

Lineage 1^a^	Lineage 2^b^	Outgroup	K1	K2	K1-K2	K1/K2	*P*-value^c^
Vg-like, *Cimex lectularius*	Vg, *Cimex lectularius*	Vg, *Pediculus humanus;* Vg, *Nilaparvata lugens*	0.643	0.360	0.283	1.79	1.0 x 10^−7^
Vg-like, *Cimex lectularius*	Vg, *Apolygus lucorum*	Vg, *Pediculus humanus;* Vg, *Nilaparvata lugens*	0.648	0.330	0.318	1.96	1.0 x 10^−7^
Vg-like, *Cimex lectularius*	Vg, *Nesidiocoris tenuis*	Vg, *Pediculus humanus;* Vg, *Nilaparvata lugens*	0.639	0.336	0.303	1.90	1.0 x 10^−7^
Vg-like, *Cimex lectularius*	Vg, *Trigonotylus caelestialium*	Vg, *Pediculus humanus;* Vg, *Nilaparvata lugens*	0.656	0.407	0.249	1.61	2.1 x 10^−7^
Vg-like, *Cimex lectularius*	Vg2, *Triatoma infestans*	Vg, *Pediculus humanus;* Vg, *Nilaparvata lugens*	0.647	0.354	0.293	1.83	1.0 x 10^−7^
Vg-like, *Cimex lectularius*	Vg, *Lethocerus deyrollei*	Vg, *Pediculus humanus;* Vg, *Nilaparvata lugens*	0.613	0.360	0.253	1.70	1.5 x 10^−7^
Vg-like, *Cimex lectularius*	Vg, *Riptortus pedestris*	Vg, *Pediculus humanus;* Vg, *Nilaparvata lugens*	0.666	0.499	0.167	1.33	2.8 x 10^−4^
Vg-like, *Cimex lectularius*	Vg, *Geocoris pallidipennis*	Vg, *Pediculus humanus;* Vg, *Nilaparvata lugens*	0.659	0.509	0.150	1.29	1.2 x 10^−3^
Vg-like, *Cimex lectularius*	Vg1, *Plautia stali*	Vg, *Pediculus humanus;* Vg, *Nilaparvata lugens*	0.637	0.522	0.115	1.22	0.014

^**a**^ Estimated mean distance between lineage 1 and the last common ancestor of lineages 1 and 2.

^**b**^ Estimated mean distance between lineage 2 and the last common ancestor of lineages 1 and 2.

^**c**^ *P*-value was generated using the program package RRTree [33].

### Tissue Specific Expression of *Vg* and *Vg-like* Genes

**Fig 3:**
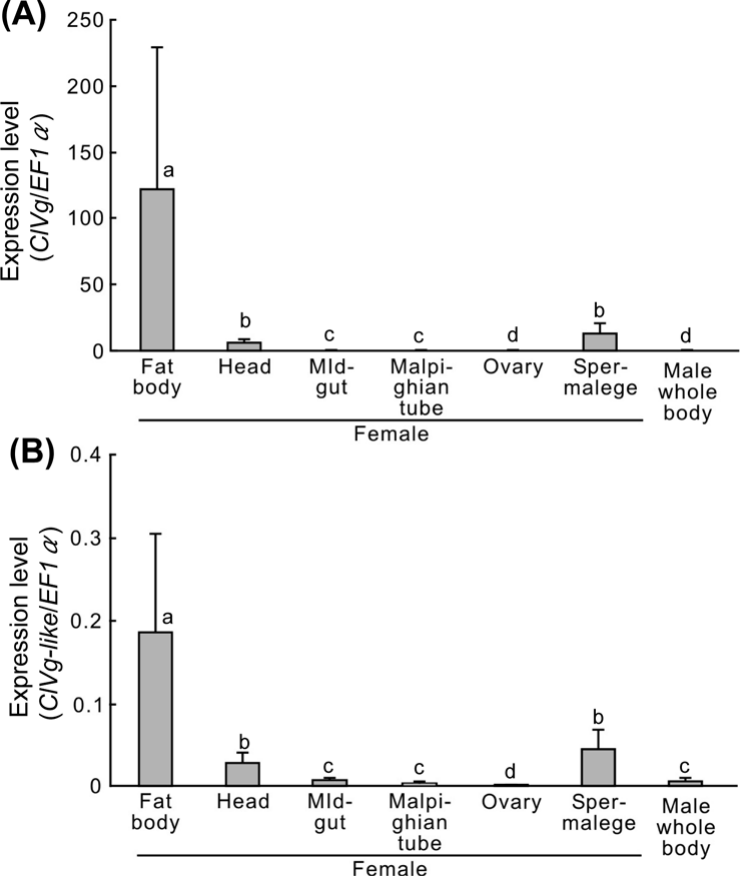
Tissue-specific expression patterns of vitellogenin genes, *ClVg* and *ClVg-like*, in the bedbug. Expression levels of the *ClVg* gene (A) and the *ClVg-like* gene (B) in dissected tissues were quantified by RT-qPCR, where expression levels of *EF1α* gene were used as an internal standard. Means and standard deviations of six biological replicates are shown. Different alphabetical letters (a-d) indicate statistically significant differences (a likelihood-ratio test of GLM and post-hoc multiple comparisons, *P* < 0.05).

We investigated expression levels of the *ClVg* gene and the *ClVg-like* gene in dissected tissues of adult bedbugs. In adult females, the *ClVg* gene was highly expressed in the fat body, whereas it exhibited a low level of expression in the spermalege, a female-specific abdominal organ involved in traumatic insemination [37] (Fig 3A). Little expression of the *ClVg* gene was detected in adult males (Fig 3A). Expression patterns of the *ClVg-like* gene were similar to those of the *ClVg* gene, but the expression levels were 10^2^−10^3^ times lower (Fig 3B). These results strongly suggest that the *ClVg* gene represents the primary functional *Vg* gene in the bedbug.

### RNAi-Mediated Silencing of *Vg* Gene Expression

**Fig 4:**
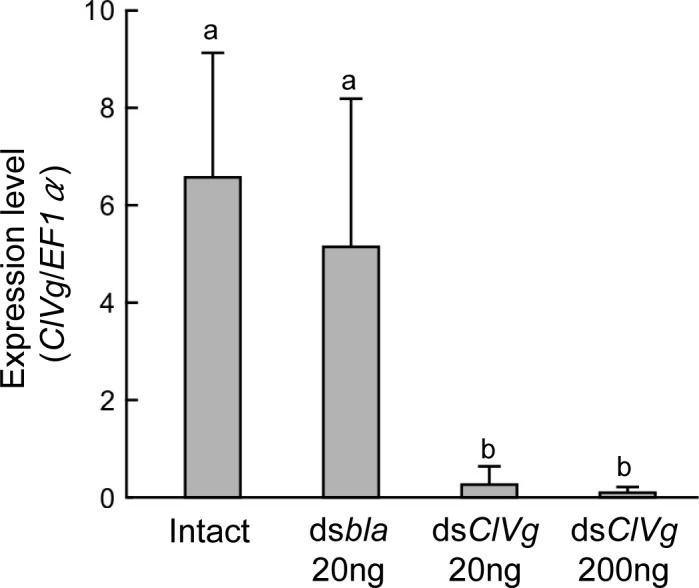
Inhibition of *ClVg* gene expression by RNAi in adult females of the bedbug. Expression levels of the *ClVg* gene were quantified by RT-qPCR four weeks after the following experimental treatments: Intact, no injection control; ds*bla* 20 ng, injection with 20 ng dsRNA of *β-lactamase* gene; ds*ClVg* 20 ng, injection with 20 ng dsRNA of *ClVg* gene; ds*ClVg* 200 ng, injection with 200 ng dsRNA of *ClVg* gene. Means and standard deviations of nine biological replicates are shown. Different alphabetical letters (a, b) indicate statistically significant differences (a likelihood-ratio test of GLM and post-hoc multiple comparisons, *P* < 0.05).

**Fig 5:**
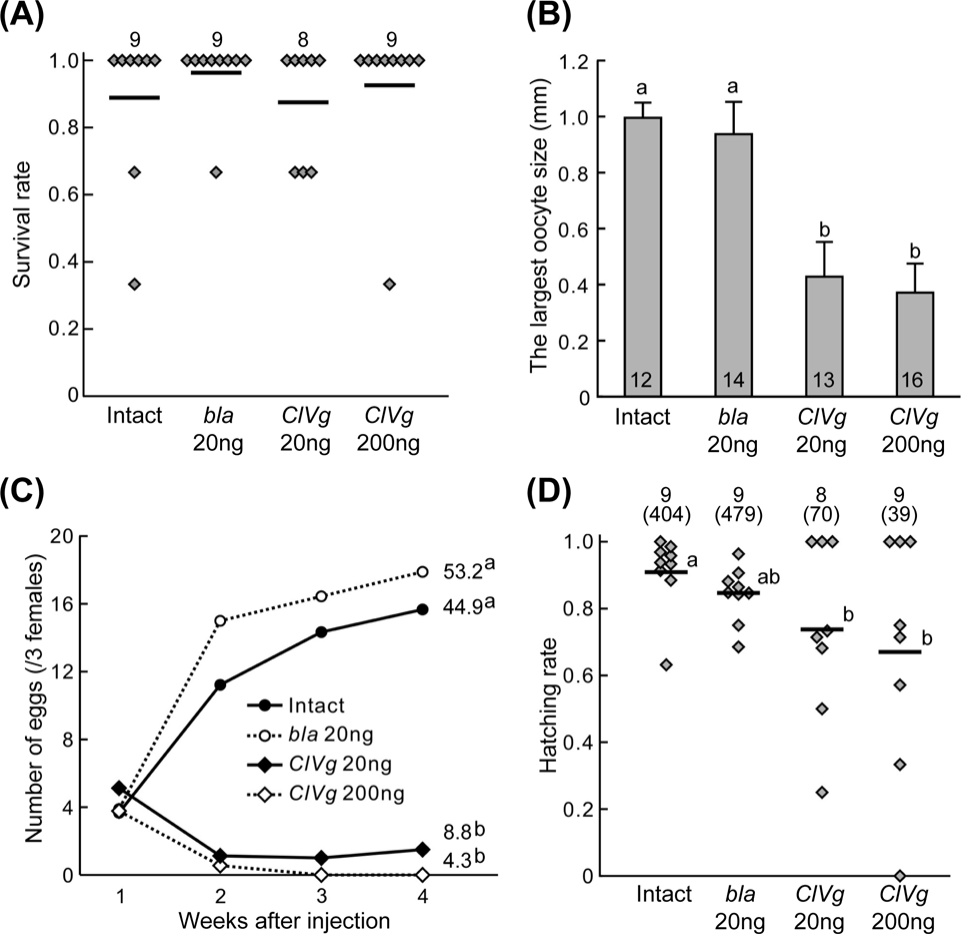
Effects of *ClVg* RNAi on reproductive performance of adult females of the bedbug. (A) Survival rates after dsRNA injection. Numbers of replicates each containing three adult females are shown at top. Rhombic points and bars indicate survival rates of individual replicates and their means, respectively. (B) Effects on oocyte size. The longitudinal length of the largest oocyte in the ovary was compared among the treatment groups. Means and standard deviations are shown. Numbers of adult females inspected are shown at the bottom of the columns. (C) Effects on oviposition. Mean egg numbers of 8–9 replicates each containing three females are shown. Mean accumulative egg numbers for the treatment groups are indicated on the right side. (D) Effects on egg hatching rate. Rhombic points and bars indicate replicates and their means, respectively. Numbers of replicates (and total egg numbers inspected for each treatment) are shown at top. In (A), there are no statistical differences among the treatment groups (a likelihood-ratio test of GLM, *P* > 0.05). In (B)-(D), different alphabetical letters (a, b) indicate statistically significant differences (a likelihood-ratio test of GLM followed by multiple comparisons, *P* < 0.05).

**Fig 6:**
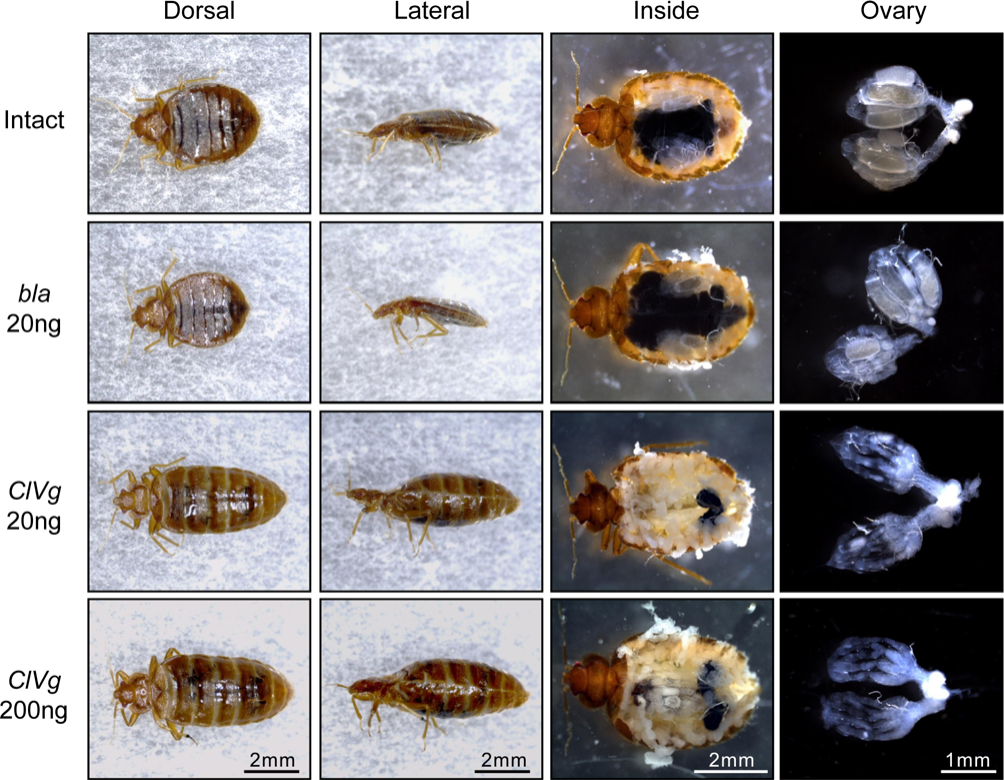
Phenotypic effects of *ClVg* RNAi on adult females of the bedbug. The insects were photographed and dissected four weeks after the following experimental treatments. From top to bottom: Intact, no injection control; ds*bla* 20 ng, injection with 20 ng dsRNA of *β-lactamase* gene; ds*ClVg* 20 ng, injection with 20 ng dsRNA of *ClVg* gene; ds*ClVg* 200 ng, injection with 200 ng dsRNA of *ClVg* gene. From left to right: Dorsal, dorsal view of whole insect; Lateral, lateral view of whole insect; Inside, dorsal view of insect whose dorsal plates were removed; Ovary, image of dissected ovaries.

For RNAi-mediated silencing of the *ClVg* gene expression, dsRNA targeting the *ClVg* gene was injected into the hemocoel of adult females at the dose of 20 ng or 200 ng per insect. Expression levels of the *ClVg* gene were drastically suppressed even four weeks after the dsRNA injection (Fig 4). During the experimental period of four weeks, mortality rates were generally low (4–12%), and survival rates were statistically not different among the treatment groups (Fig 5A). On the other hand, the *ClVg* dsRNA injection resulted in remarkable phenotypic consequences. The abdomen of adult females subjected to the *ClVg* RNAi was conspicuously swollen like fully-engorged bedbugs (Fig 6, “Dorsal” and “Lateral” columns). The swollen abdomen of the *ClVg* RNAi females was full of hypertrophied fat bodies, which was in contrast to much less developed fat bodies adhering to the inner wall of the thinner abdomen of the control females (Fig 6, “Inside” column). In the *ClVg* RNAi females, ovaries were atrophied with no mature oocytes, which were in contrast to well-developed ovaries containing mature oocytes in the control females (Figs 5B and 6, “Ovary” column). In the *ClVg* RNAi females, egg production was drastically and significantly suppressed in comparison with the control females (Fig 5C). Notably, in all eight replicate groups (three females each) injected with 200 ng dsRNA, and six of eight replicate groups injected with 20 ng dsRNA, females completely ceased egg production two weeks after the dsRNA injection. It is also notable that eggs produced by the *ClVg* RNAi females tended to suffer low hatching success (Fig 5D). Taken together, it was concluded that RNAi targeting the *ClVg* gene effectively inhibited the bedbug reproduction.

## Discussion

Vg proteins are the major yolk precursor proteins whose structure and function are conserved among diverse insect species, although there are some diversity in number of cleavage sites, number of duplicated genes, and tissue-specific expression patterns [6,7]. In this study, we identified two *Vg* genes, *ClVg* and *ClVg-like*, in the bedbug (Fig 1). Some insects possess multiple *Vg* genes, whose sequences are usually highly similar to each other with retaining conserved functional domains and participating in vitellogenesis [38–41]. However, the two *Vg* genes of the bedbug are considerably different in their sequences. The *ClVg* gene exhibited canonical insect Vg protein structures including RXXR cleavage sites and conserved binding sites for a variety of nutrients (Fig 1A), and its transcription was highly up-regulated in the fat body of adult females (Fig 3A). RNAi-mediated silencing of the *ClVg* gene expression confirmed its primary contribution to oocyte maturation (Figs 5 and 6). These results indicate that the *ClVg* gene encodes the principal Vg protein involved in the conventional vitellogenic function in the bedbug. On the other hand, the *ClVg-like* gene lacked the N-terminal lipid-binding region (Fig 1B), although the *ClVg* gene and the *ClVg-like* gene are presumably derived from the common ancestral *Vg* gene (Fig 2). Since the *ClVg-like* gene still retains putative cleavage sites and some domains typical of insect *Vg* genes (Fig 1B), the possibility cannot be ruled out that the ClVg-like protein may be subjected to posttranslational processing and also incorporated into the oocytes as typical Vg proteins. However, expression levels of the *ClVg-like* gene in the fat body were incomparably lower than those of the *ClVg* gene (Fig 3), refuting the possibility of substantial contribution of ClVg-like protein to vitellogenesis. Considering its structural divergence (Fig 1) and accelerated molecular evolution (Table 2), it is conceivable, although speculative, that the *ClVg-like* gene might have acquired a novel biological function distinct from the vitellogenic role of the *ClVg* gene in the evolutionary course of the bedbug. In this context, previous studies suggested that Vg proteins may also be involved in hemolymph clotting [8,42], hormonal regulation [43], innate immune responses [44], and other biological roles. Further studies are needed to address what roles the *ClVg-like* gene plays in the bedbug.

Although the main location of Vg protein synthesis is the fat body in diverse insects [6,7], there are several reports on Vg protein production outside the fat body. In a blood-sucking reduviid bug *Rhodnius prolixus*, Vg protein is partially synthesized in the follicle cells, which is also incorporated into the oocytes together with the major Vg protein derived from the fat body [45]. In a blood-sucking tick *Haemaphysalis longicornis*, one *Vg* gene is exclusively expressed in the midgut, the other two *Vg* genes are mainly expressed in the fat body, and all the three genes are involved in oocyte maturation [21]. In the bedbug, we found that the *ClVg* gene is also expressed in a female-specific paragenital organ called the spermalege (Fig 3). The spermalege is a pouched mesodermal tissue attached to the abdominal inner wall of adult females of cimicid bugs [37], whose function is postulated as a counter adaptation against the traumatic insemination, a peculiar reproductive habit typical of cimicids [46,47]. Although the possibility that the ClVg protein synthesis in the spermalege may somehow contribute to vitellogenesis cannot be ruled out, a more likely possibility is that the ClVg protein may be recruited as a clotting element in this organ [8,42], considering that the spermalege is the site of frequent wounding by traumatic insemination.

We found that RNAi-mediated silencing of the *ClVg* gene expression effectively inhibits egg production in the bedbug (Figs 5 and 6). The significant inhibitory effects on egg laying became evident two weeks after the dsRNA injection (Fig 5C). The initial egg production is likely attributable to the Vg protein accumulated before the dsRNA injection. Note that we performed the dsRNA injection two days after blood meal, and the bedbugs normally start laying eggs three days after blood feeding [48]. It is also notable that the eggs produced by the *ClVg* RNAi females tended to suffer low hatching success (Fig 5D), which is also likely attributable to insufficient accumulation of the Vg protein during oogenesis. Furthermore, all females injected with 200 ng dsRNA and most females injected with 20 ng dsRNA completely stopped laying eggs two weeks after the dsRNA injection, in which no mature oocytes were found in the ovaries (Figs 5 and 6). On the basis of these results, we propose that the *ClVg* gene can be a promising target for RNAi-based control by inducing reproductive arrest of the bedbug.

We also found that abdominal inflation is another remarkable symptom of the *ClVg* RNAi in the bedbug. The external appearance of the insects looked like that just after full engorgement, but their abdomen was actually full of hypertrophied fat bodies instead of a blood-filled stomach (Fig 6). Such a phenotypic syndrome associated with *Vg*-RNAi was not observed in the cockroach *Blattella germanica* [20] and the tick *Haemaphysalis longicornis* [21], although in the latter species the Malpighian tubules and the rectal sac were abnormally filled with white liquid [21]. The peculiar abdominal inflation may be relevant to feeding habit of the bedbug. Adult bedbugs ingest blood about two times of their own weight in a single meal by expanding intersegmental membranes of the abdomen, and the acquired nutritional resource is equivalent to production of 15–19 eggs [48,49]. The *ClVg* RNAi may inhibit not only the synthesis of Vg protein in the fat body but also the transportation of associated nutrients from the fat body to the oocytes. Conceivably, nutritional resources derived from blood meals are stagnated in the fat body, thereby resulting in its hypertrophy and consequent abdominal inflation.

In addition to the nutritional stagnation, the abdominal inflation due to the *ClVg* RNAi may influence the pest status of the bedbug by way of its behavioral modifications. First, because of the stuffed abdomen, the insects may have little room for further blood ingestion (see Fig 6), thereby presumably suppressing their blood-sucking activity. Second, considering the fact that fully-engorged female bedbugs frequently receive traumatic insemination because their swollen abdomen makes it difficult to take a guard posture against sexually aggressive males [37], it seems likely that the *ClVg* RNAi females with the inflated abdomen may suffer continuous and repetitive harassment by conspecific males. It was reported that accumulated traumas reduce female longevity not only by damage of integument piercing but also by increasing risks of microbial infections [46,47,50,51]. Experimental verification of these behavior-mediated effects of the *ClVg* RNAi deserves future studies.

The recent global resurgence of bedbug populations with resistance to broad-spectrum chemical insecticides has prompted exploration of new pest management strategies [12]. Our study demonstrated that an RNAi approach targeting the *ClVg* gene causes drastically suppressed egg production and remarkable abdominal inflation in adult females of the bedbug, which possibly reduces reproduction, feeding frequency and longevity of this notorious pest. These findings suggest the possibility that the *ClVg* gene is a promising candidate for RNAi-based population management of the bedbug, especially in an early phase of population growth on account of its non-acute effects on the insect reproduction. What needs to be solved for enabling practical applications is the development of technologies for efficient delivery of dsRNA into the bedbugs. Microinjection into individual insects is practically not feasible. The effectiveness of oral ingestion of dsRNA has been reported in several hemipteran insects including the blood-sucking reduviid bug *Rhodnius prolixus* [52–54]. For agricultural insect pests, application of transgenic plants that produce dsRNA of target insect genes has been attempted [55,56]. For blood-sucking insect pests, however, using transgenic animals for supplying dsRNA-containing blood meal is unrealistic both practically and ethically. Therefore, future studies should be directed to the development of alternative dsRNA delivery methods like chemo-attractive feeding traps [57] and microbial vectors [58,59], in parallel with survey of other target genes for more efficient control of bedbug populations.
